# Making as Method in Teaching: Do-It-Yourself (DIY) Objects and Hands-on Learning with Materials

**DOI:** 10.5334/pme.1575

**Published:** 2025-05-20

**Authors:** Anna Harris, Martina Bardelli, Giuliana Brancaleone, Nyah Costa, Lia Hruby, Remco Poeliejoe

**Affiliations:** 1 Department of Society Studies, Faculty of Arts and Social Sciences, Maastricht University, NL

## Abstract

In medical education, technological innovation often focuses on the digital and virtual. In the analogue space, physical learning tools seem to come readymade – pre-programmed mannequins, printed textbooks or the ubiquitous articulated plastic skeletons. The market for mass-produced objects in medical education is vast, however we concern ourselves here with important but overlooked learning materials that fall outside this digital-industrial complex: handmade objects, crafted using (often) simple, low-cost, locally sourced materials, also known as *DIY objects*. Educational materials have long been hand-crafted, yet this topic receives little attention in the healthcare professions education literature. In this Eye Opener article, we aim to bring DIY objects out of the shadows and in doing so, introduce to the healthcare professions community some of the main theories, movements and approaches behind making as a teaching method. To further our understanding of the role of DIY objects in medical teaching we adopted an ethnographic method that involved making the objects ourselves. Our Eye Opener suggests a greater emphasis can be placed on making one’s own teaching materials and on making as a learning activity. We discuss how making facilitates active and multisensory modes of learning including enhancing spatial awareness, helps students to challenge the status quo in medicine and encourages environmental sustainability in the classroom. We propose some applications of making in the classroom, such as exploring more diverse representations of bodies and studying the environmental impact of medical education materials.

## Introduction

The cognitive basis of producing and learning medical knowledge has been well established in the field of medical education, through psychologically informed research on topics such as deliberative practice [1], decision-making [2], problem-solving [3] and more recently including the emotional [4] and identity-based [5] nature of medical work. Recently however, researchers in medical education, especially those using qualitative research techniques such as ethnography and interviewing, have expanded the focus to encompass the more bodily, sensory, social and material nature of medical learning, thereby strengthening the recognition of the sociomateriality of learning [6]. Reflecting trends in the broader social sciences and humanities, medical educators are now expanding on sociomateriality by moving beyond text, exploring drawing [7], photography [8] and poetry [9] in the classroom, amongst other methods.

In this Eye Opener paper, we expand on the existing sociomateriality literature to explore the role of *making* in medical education. Our focus on making highlights an often taken-for-granted aspect of medical learning, which is the role that physical objects such as models, mannequins, books and charts, etc. play in how medical knowledge is taught and reproduced.

Our article has been informed by comparative and collaborative ethnographic research, conducted by a group of researchers and students in the Netherlands about the role of materials in medical learning. Throughout this research, ethnographers and historians explored the instances and possibilities of expanding making in their field sites. The ethnography was conducted over the course of seven years, involving fieldwork in four different medical schools in four countries (The Netherlands, Ghana, Hungary and Australia), and culminated in a series of hands-on workshops and interventions designed and led by students in the Netherlands. The results of this larger ethnographic research project called Making Clinical Sense, which have been published elsewhere (see references listed on the project website www.makingclinicalsense.com), documented the lively practice of handmaking materials in medical schools around the world. For this article the authors conducted additional experiments to extend the insights of the broader study, bringing making methods front and center in the classroom. This is a topic that has not been previously explored in health professions education to our knowledge. Before we describe the insights we derived from our experiments, we introduce the Making approach, the importance of materials, and medical education as a context for making. Our overarching goal with this paper is to inspire others to experiment with making in their own practice, whether it be teachers or students.

## Making approach, with a capital M

Making is an everyday term which has been adopted differently by various communities and movements. In many communities, making has a capital M, as in: Making. Here Making is described as a series of activities focused on designing, building, modifying, and/or repurposing material objects for playful and/or useful ends [10]. This definition of Making draws from what is known as the “Maker movement”, which originated in the United States and describes the broader cultural movement around Making, including in education [11,12]. The Maker movement has pushed a specific “learning by doing” approach, where learners become Makers of physical solutions to local and immediate problems [10]. Thereby, learning by doing is often seen as actively engaging with materials or tasks to acquire knowledge through experience, rather than passive instruction. There is a focus here on materiality that relates but is different from other “learning by doing” scenarios medical educators might recognize. For example, in simulation-based learning, learners may engage in highly controlled, realistic scenarios where they apply theoretical knowledge to solve a specific problem, whereas in Maker education there is more open-ended exploration and iteration with objects. Learning by doing in a clinical setting might allow for more unpredictable, real-world conditions where learners interact with patients while adapting to diverse situations. In the context of the Maker movement, learners engage in making physical objects, often without predefined outcomes and involving continuous problem-solving. This kind of engagement fosters forms of adaptability and creativity that are harder to cultivate in environments with fixed parameters [10].

Making has gained significant attention within science, technology, engineering, and mathematics (STEM) curricula. Education researchers have examined a variety of potential benefits that arise from incorporating Making into these disciplines [11,12]. These include fostering creativity, enhancing problem-solving abilities, and providing students with opportunities for active, experiential learning. Additionally, studies have shown that engaging in Making helps students build persistence, as they navigate iterative processes of trial, error, and refinement. Making also strengthens collaboration and communication skills, as many projects involve working in teams. Besides gaining knowledge, Making helps students develop a sense of identity as creators and innovators – characteristics that are highly valuable in numerous fields, including education, technology, and healthcare [11,12]. This understanding and value creation of Making reflects a broader situatedness within engineering and scientific discovery. As the movement evolved, it has gained increasing recognition beyond a tool for creativity and play but also as a legitimate approach to education more generally. This shift becomes evident in the proliferation of Makerspaces for children as well as university students, which include technological infrastructures such as 3D printers, podcasting equipment, and laser cutters.

Researchers focusing on Making in education propose hand-making artefacts and hands-on learning as an active and effective way of keeping students engaged in their learning processes [13,14,15,16,17,18]. This way of learning emphasizes the role of the student as an active participant in knowledge construction, as described in learning theorist Seymour Papert’s theory of *constructionism* [19]. Making in learning is underpinned by constructionist theories, suggesting that learners construct knowledge more effectively when engaging in the creation of tangible artefacts in the real world [17,18]. Constructionism places a strong emphasis on hands-on, project-based learning [19], proposing that the creation of meaningful designs leads to the internalization of actions [18]. Such theoretical principles are foundational to constructivist pedagogical approaches in education from the Maker movement [20,21,22]. Now having reviewed some of the main claims of Making in general, we move onto the role of materials in this approach.

## Making *with* materials

An important element of the Maker movement is also the *Do-it-Yourself (DIY)* culture, which revolves around the use of accessible, low-cost materials to construct new objects for use [23]. Through the Maker movement, educators and learners highlight the value of material experimentation in DIY-oriented projects as an effective means of learning. To expand an understanding of the role of making in healthcare education, teaching and learning, it is helpful to examine theoretical ideas concerning materiality, from fields such as arts education and anthropology.

Within these fields making is framed as a social and bodily practice whereby the learner engages with the properties of materials, and learns *with* them, not *about* them. The embodied nature and the materiality of the novice’s engagements are fundamental to what is learned. Such an approach to learning can align with theoretical traditions such as embodied cognition, which would situate learning in a broader distributed arrangement, including objects and places, or with the pragmatist theories of John Dewey, who argues that in learning people make themselves. While these theories are connected to our arguments, in this article we chose specifically to draw from the work of cultural anthropologist Tim Ingold [24].

In his many books and articles, Ingold suggests that learning is a bodily correspondence *with* materials, resituating learning away from a more cognitive form of knowledge transmission where learners find out *about* materials and other things or processes. This might involve a student not only reading about the history of the stethoscope as a rolled-up tube of paper but also making one themselves [25]. Learning with materials is a sensory engagement with the world of things. The senses are important here as a bedrock to how learning happens in life more generally, brought into the university where it is so often neglected [26]. Through using touch, movement and hearing for example, students can activate different ways of learning than the current emphasis on sight through reading and writing. Ingold [24] has expanded on these ideas in practical ways through lessons he has developed which involve students making kites and baskets to learn about social theories in anthropology. His work also correlates closely with the study of crafts by sociologists and anthropologists such as Richard Sennett [27] and Trevor Marchand [28], and has also been expanded by Science and Technology Studies (STS) scholar Kat Jungnickel [29].

Making as an anthropological concept also expands on the cognitive approaches to learning by forwarding the idea of “material thinking” [30]: a thinking through the hands, in correspondence with things, as a form of self-discovery. The idea of material thinking has been forwarded in fields such as architecture (for example in learning through making models) as well as art and anthropology (e.g. through the craft lessons we described above but also in much of arts education).

Our ethnographic research was extensively informed by Ingold’s work on making and the idea of material thinking. For instance, in our experiments, we tried to learn how to make teaching tools ourselves in a workshop setting. We created a model of an ear by following an online instruction video and using the materials suggested, while noting observations of our sensory workings and learning. Anna Harris has written further about ways medical teachers improvise with the objects they have at hand, with Jan-Joost Rethans [31], however we wanted to expand on this further, in dedicated experiments. Few universities currently facilitate making or DIY-based projects as a learning activity in the medical field and very little has been written about these activities as a pedagogical approach. In the next section, we present the available literature and outline what is known about these practices in medical education.

## Making in medical education

When it comes to objects in the classroom, medical learning still relies heavily on expensive industrially produced models [32,33]. This reliance on standardized models, however, overlooks the homemade in medical education. Historically, medical education is built on making and experimentation, driven by different factors – necessity, pedagogical innovation and resource constraints [34]. From the early days of apprenticeship-based learning to structured institutional learning in the contemporary classroom, educators have relied on physical models and representations to translate the complexities of the human body into more simple, tangible, and teachable forms [35]. Take for example, the models and other representations of bodies developed by educators to recreate the human body for students in simulated environments. On top of their advantages for teaching, making these anatomical models reflects active learning processes. Schmidt, Thompson and Chang [36] described that as students made pelvic models in clay, their confidence and abilities to share their takeaways with other students increased. These handmade medical models have varied in material forms from wax, papier-mâché, clay, yarn, pop-up books, plastic body parts, collages, mannequins with speakers and leaking orifices [37].

Although the handmade has become less visible in medical schools, our ethnographic research showed, and other anthropologists have documented, that locally produced models are still made and used [38,39,31]. These homemade materials are shared locally and occasionally published in research papers or blogs [40]. In her work on simulation education in Ghana, STS scholar Andrea Wojcik [41] writes that using simple, affordable materials, means that schools can function without always acquiring newer technologies, and it invites “teachers and curriculum designers to imagine other possible simulations that might address the shortcomings of inherited simulation practices as well as promoting a return to older collaborations between medicine and art”.

We also consider the local nature of making practices in medicine important and consider it a pity that making is not more widely discussed, recognized and shared in medical education, especially considering its potential as an educational practice, as outlined in STEM fields, art and anthropology. In the following section we explore ideas for incorporating making in medical education based on a series of making experiments we have conducted.

## Making experiments in medical education

In previous sections, we shared examples of making in medical education and now we would like to practice what we preach and explore the topic further, in a hands-on manner. Our experiments in this article are informed by previous work done in the context of the broader ethnographic study Making Clinical Sense. Making Clinical Sense involved workshops with medical educators with simple materials from the craft store, that explored their own pedagogical solutions to teaching clinical skills, or knitting a uterus that was regularly used in obstetrics lessons, recreated with a pattern found in medical archives. These examples revealed the processes teachers go through to consider the affordances (i.e. possibilities) of different materials. Making teaching objects provided bodily insight into the process of material selection and the decisions made in the object’s construction.

Following this work, we, the authors of this article, comprising a teacher and students in an arts program, undertook a four-month-long series of experiments exploring how DIY models could be further incorporated into the learning processes in medical education. The data collection for this project consisted of four distinct sources; designing and observing group workshops involving making medical teaching tools; unstructured interviews with medical students and teachers from the medical faculty; field visits to medical schools and observations in medical classrooms of existing mannequins; and video and photographic documentation of the making workshops and materials used.

**Figure 1:**
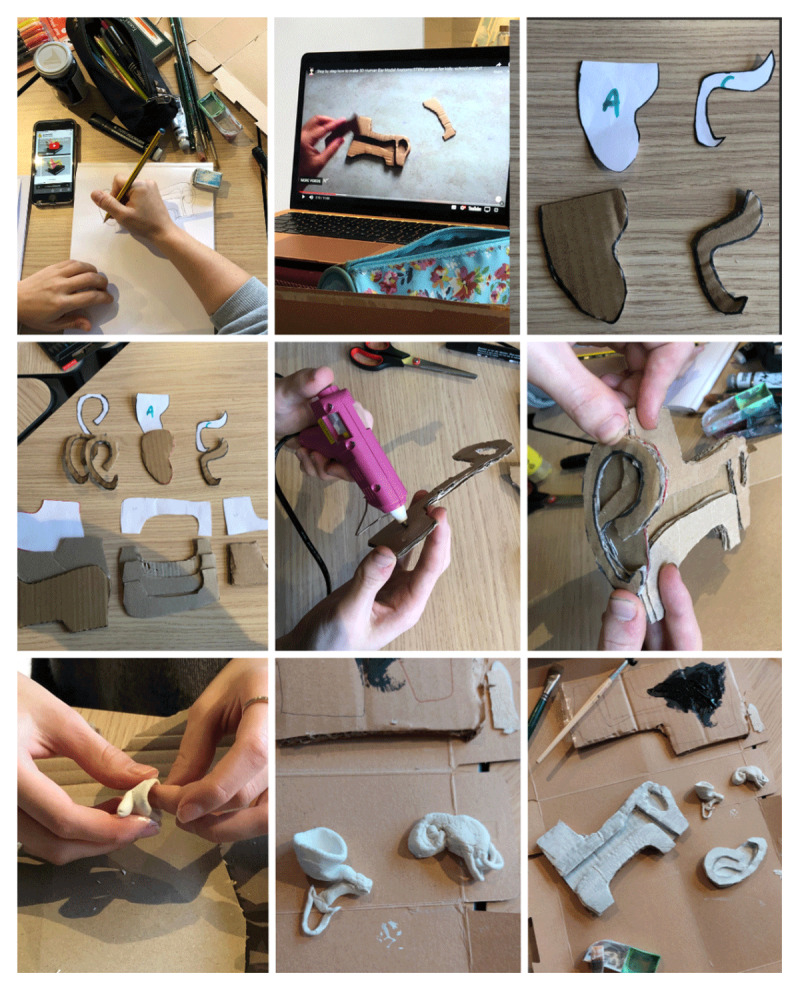
Story board of our group making the 3D ear model, student-led.

**Figure 2:**
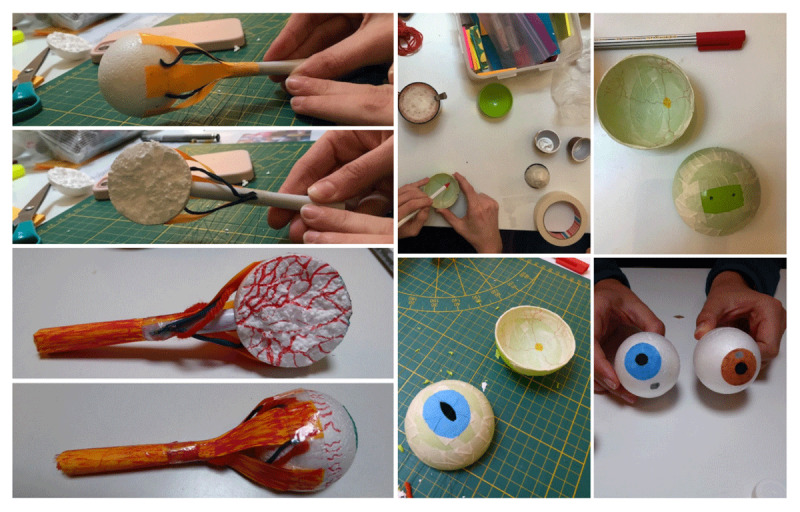
Students from our group making 3 different 3D eye models (visualizing its anatomy, vessels and potential spottings), led by Marijke Kruithof.

Here we outline two lessons as examples of bringing making into the classroom. In this case it was an arts classroom, however it also applies to medical schools. In two separate workshops, our group recreated a 3D model of the human ear following instructions in a video (Figure 1), and several 3D models of the human eye found in an anatomy book (Figure 2). Both workshops began by comparing prior knowledge of relevant anatomy. Then, we consulted the sources together and assigned different model components to participants, who researched the function of their component before physically crafting it. For example, when making the cochlea for the ear model (Figure 1), a student researched its role in the auditory system and shared it with the group. This integration of research and making encouraged active and engaged participation, and knowledge exchange, reinforcing the relationship between material construction and conceptual understanding. Making in these workshops significantly differed from a passive knowledge transmission model, such as a lecture, in which students are *given* knowledge, instead of *making* it. We concluded our workshops by presenting the models, reflecting on the learning process and comparing new insights to initial anatomical knowledge. By physically shaping anatomical structures, everyone reported an improved spatial and functional understanding of complex forms – an approach that has clear potential for medical education. We documented the process through time-lapse videos, photographs, field notes and drawings, while reflecting together and individually on the educational value of making the objects we crafted.

## The benefits of making for learning

From our experiments we identified three main benefits of making as a hands-on approach for learning in medical education:

1. *Multisensory learning:* Our making projects involved feeling, smelling, touching, palpating, glueing, watching and listening. We see the making of teaching models as a contribution to the recent return to emotional and sensory learning methods in the learning sciences, that has become more salient in the context of the heavy investment in digital learning tools. Amidst this emphasis on the possibilities of the digital, which has exploded since the pandemic, learning scientists have urged that we must not neglect the importance of embodied learning [42]. Making entails, Ingold reminds us, what the ecological psychologist James Gibson calls an education of attention [24], meaning that the novice must pay attention carefully to what something feels like, looks like, sounds like, and the potential uses and possibilities of the materials.
2. *Sustainability:* Our models and making involved using low-cost materials we found around the home. Learning medicine through the making of DIY objects using simple and locally available materials offers opportunities to create more locally relevant and affordable learning models with material alternatives. Making orientates the learners towards the materials that are available around them, and their generative currents, rather than reaching for the ready-mades on the shelf or relying on the teacher to supply what is needed. Making also involves learning from the materials. It means that students start to consider the histories and localities of what they are working with in their own educational setting.
3. *Diversity:* The effects of colonisation remain prevalent in medical education today [43,44] shaping medical knowledge through the imperial spread of learning materials. These effects are best reflected in the donations of medical teaching objects and the funding of research plans to the Global South [43,45]. Through field site visits, most of the authors of this paper who were at the time arts students and who were involved in the experiments highlighted the overrepresentation of white heteronormative abled bodies in the mannequins stored in the medical school. They brainstormed on the opportunities of making alternative models in the workshops. Interestingly, the arts students’ interviews with medical students revealed limited reflection by the medical students on these elements of diversity. These views indicate some degree of limited questioning of the white, heteronormative narrative, but also its reinforcement through the absence of underrepresented bodies. Thus, making models can also work towards challenging the status-quo in medical learning settings by incorporating critical thinking, engaging students more actively with their learning materials, and finding alternative and inclusive ways of depicting human bodies. This learning method would allow the students to be more actively engaged with the different kinds of bodies represented in their curriculum and local communities. Thus, making enables a constructionist approach to learning but also offers the potential for intervening in the broader social and post-colonial context of learning.

## More making in medical education

We suggest that HPE educators use these benefits for making in learning to incorporate more making in the classroom. The making approach also has potential to shift dominant trends in medical education. Currently, there is significant interest and investment in industrially produced models and technologies which emphasise corporeal fidelity and aesthetic sophistication. This is an unsustainable model in the longer term. We can only start to alter current practices by addressing the material and cyclical replication of it. Making could add depth to topics related to diversity, inclusivity, physical spaces of learning, history and other themes.

**Figure 3:**
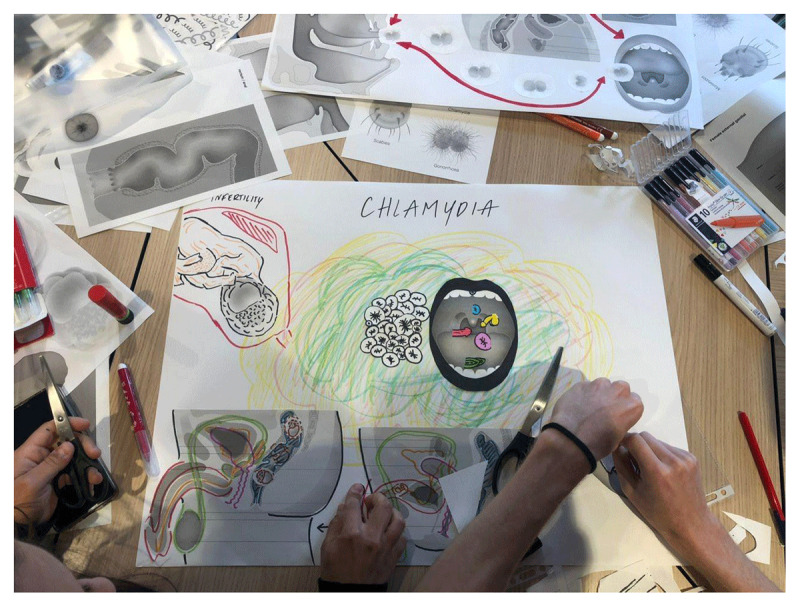
Our group learning on the modes of transmission of Chlamydia as part of an STI creative workshop led by Kuang-Yi Ku.

Making in the classroom presents challenges which reflect broader issues encountered in creative teaching and research methods. Making involves educators selecting models that align with course content while ensuring they are not overly time-consuming for students to create. Interviews with medical students have revealed that they would prefer engaging in making when the initiative comes from the teachers, as DIY can feel *intimidating*, but also to be certain of the validity and quality of the objects created. Additionally, finding affordable, widely available materials across different locations can be a barrier, as can securing access to suitable teaching spaces that accommodate hands-on work. Nonetheless, not all DIY models require specific materials, extensive preparation, nor simulation labs. For example, Figure 3 depicts a workshop on STIs conducted as part of our experiments and led by a collaborating artist. The model shown visualizes the modes of transmission of chlamydia and only required paper, scissors, glue and colored markers.

Another key challenge is assessment. How should educators evaluate the outcomes of students’ making projects? Beyond grading, questions arise regarding how to document, share, and standardize DIY learning models, especially when they exist outside formal curricula. Similarly, intellectual property and ownership concerns emerge: Who owns the rights to a model created by students in a classroom setting, and how can such resources be shared fairly within – and outside of – the field? Perhaps the Open Educational Resources (OER) movement can offer solutions to this challenge. OER is defined by the UNESCO as educational materials produced in various formats that are publicly available, with an open license, which permit freeaccess, and allow for re-use and redistribution [46]. There is a great deal to learn from this movement, in uncovering how learning materials are shared and acknowledged worldwide, as well as learning from post-colonial medical education theorists, like Alan Bleakley, Julie Brice, John Bligh, and Thirusha Naidu, who question the inclusivity of these efforts and the dominance of Western medicine in medical education curricula worldwide [43,44].

To explore further solutions to the latter challenges, we are currently piloting a project whereby we gather medical DIY models from various sources (research papers, OER, online videos), and produce both an analogue and digital catalogue of DIY materials made and used in medical education. This catalogue aims to raise further awareness on the importance of making as a method in medical teaching and learning, but also to shed light on existing models and knowledge, which to date are largely restricted to local communities. We believe this can enable greater learning opportunities in medical education worldwide, as special attention is also given to material availability and affordability in different contexts and locations. The catalogue strives to maintain the sensory richness of learning with locally relevant materials and urges for greater diversity and representation in making processes. The objects selected are often described through interviews with makers from the field or through reviews by medical students, as one possible way to navigate intellectual property challenges. The catalogue will be formatted as a black and white PDF so that it can be printed and shared for free in physical form, as well as in an open-access digital format to reach a wider audience. The catalogue is just one effort towards the challenge we see of sharing educational resources within – and outside of – the medical field and existing local communities, reflecting the purpose of the Open Educational Resources movement.

## Inspiration and conclusions

The findings of our experiments highlight the possibilities of making as a method, a method that has long underpinned the training and teaching of healthcare professionals. In summary, we suggest that making is a currently underutilised but insightful method for teaching in medical education.

Our own investigation into this topic ultimately ended up offering surprising insights into many norms of medical education from the prevalent use of expensive and non-representative models, to the rise of digital learning tools. In our making workshops, we saw how learning through making DIY models allowed for the use of local, low-cost materials and thus, offered a more affordable, adaptable and sustainable method of teaching and learning medicine. Further, they provided students with the opportunity to explore alternatives to the prevailing norms associated with how the body is represented in medicine, questioning established modes of knowledge reproduction in such fields. Moreover, we showed that engaging in learning through making plays a crucial role in active multisensory modes of engagement in the classroom, especially in regards to spatial awareness. It also accentuates the students’ role as contributors in constructing their own knowledge, which opens ways to consider how learning experiences might be otherwise. Building on anthropological and STS perspectives on making, we believe in the importance of maintaining (multi)-sensory learning approaches, working with physical materials and using DIY models and approaches in physical spaces as teaching methods in the midst of the ongoing digital transformation.

We have argued that making methods are a generative way of incorporating and attending to the role of embodiment and the senses in learning processes. Of course, making should never be used as an end to itself, simply because it is innovative or fun, but rather it should be employed as a means to an end: to help reach specific learning goals. We hope our work serves as a catalyst for further making in classrooms, thereby enabling innovative learning approaches that bring sociomateriality further to the fore in HPE in new ways.
